# Transactions of the Chicago Gynæcological Society

**Published:** 1879-01

**Authors:** W. H. Byford


### Society Reports.

### ARTICLE IX.

### TRANSACTIONS OF THE CHICAGO GYNÆCOLOGICAL SOCIETY.

SECOND MEETING, NOVEMBER 29TH, 1878. W. H. BYFORD, M. D., in the chair.

DR. SAWYER reported a fatal case of puerperal inversion of the uterus (published in the present number of the JOURNAL AND EXAMINER).

DISCUSSION.

DR. MILLER: The subject introduced this evening by Dr. Sawyer, is one of more than ordinary interest to every accoucheur. The report of the case is admirable, and the specimen presented adds greatly to its value. I have no criticism to offer in regard to the management of the case, for it was faultless. The accident happens but seldom. It was fortunate that in this case it occurred under the immediate observation of one so competent to comprehend its gravity from the initial stage, and who could present to us so clearly its complete history.

The causes of inversion of the uterus are various — generally, I believe, mechanical; not always so, however. I can appreciate that inversion may take place as follows: The uterus in a state of complete inertia, from long continued labor, from too sudden delivery of the child, or from over-distension, as in Dr. Sawyer's case, a slight cause may produce depression and end in inversion. That cause may be the increased weight of that portion of the uterus upon which the placenta was developed, if near the fundus, together with the other condition which obtains at the site of placental development, namely, the projection inward of that part of the uterus. The structure of the uterus, immediately surrounding the placental site, in the condition assumed, could offer no resistance to the further settling internally by gravity of this heavy circumscribed portion of the uterus — depression having occurred, inversion would follow.

Another possible cause of inversion may be mentioned, though not strictly applicable to the case reported this evening. When the uterus is in a state of complete inertia, and the placenta has been detached, and has lodged within the os uteri, the cavity of the uterus, as is usual in such cases, remains large, and is filled with blood. The extraction of the placenta will act as a piston upon the volume of blood in the cavity of the uterus, and this, in turn, will act upon the flaccid wall of the uterus ; the fundus may follow this forcible extraction of blood, till depression is produced. Then inversion may be completed by the uterus itself.

I am not certain that attempts at compressing the uterus may not produce a dimpling of the organ that will lead to inversion, especially if performed carelessly, or by a person not familiar with the physiology of uterine contraction. It appears to me that inversion is liable to be caused by the injudicious application of force to the uterus in a state of inertia.

DR. ROLER : The case presented for discussion is an interesting one, but the cause of the inversion cannot be determined, as the attending physician has no knowledge of the exact moment of its occurrence. The delivery of the placenta evidently had nothing to do with the accident, as manipulation immediately afterward detected the outline of the uterus in a flaccid state, the fundus being near the umbilicus. Ergot was administered, and firm pressure applied both over the uterus and upon the abdominal aorta. A little later, when contraction suddenly occurred, the cup-shaped depression was easily recognized by the hand over the abdominal parietes.

The fundal sinking must have taken place before contraction set in, as symmetrical action of all the muscular fibers would insure safety against such an accident. The condition of ergotism may have been the chief cause of failure in the attempted reposition of the uterus, but the administration of the drug was clearly indicated, and Dr. Sawyer did as any intelligent physician would have done under the circumstances.

I fully endorse the remarks of Prof. Miller with reference to careful manipulation in the removal of the placenta, even after uterine action has thrust it into the vagina. Leishman, J. Matthews, Duncan, and others, state that if left alone it usually presents edgewise during expulsion, but I think the bedside experience of every physician proves that without meddlesome interference it is often found fœtal surface foremost, and "impacted" in the vagina. Of late years I have always, when removing it, waited until I found the uterus well contracted above the pubis. It ought to be brought out edgewise and slowly. Traction upon the cord would cause a vacuum above, which would be filled with blood from the open sinuses, or if atony was present, possibly by depression of some part of the uterine wall.

In regard to the forces which operate to bring about complete inversion, I would like to state my doubts as to the ability of the organ, by any contractile power, to invert itself. I am aware that it has become an accepted doctrine that the introverted part may be seized by the remaining portion, and be carried downward, thus completing the process by its own action. I do not think it has been satisfactorily proven by any one who has written on the subject. In what way can any expulsive force be developed when the fundus is not acting? It is a matter of common experience when the uterus fails to contract symmetrically, in any stage of labor, that the expulsive process is arrested. In irregular contractions, even the detached placenta is held in vice-like grasp until artificially removed. If the introverted part contracts, it shortens itself merely. If the encircling portion acts, the further descent must certainly be arrested by the grasp of the circular fibers. Are not the incomplete varieties owing to this fact? When general atony exists, the weight of the descending portion, aided by the straining on the part of the patient, will easily carry the fundus into the vagina.

Irregular contraction is doubtless a primary condition in many cases, of incomplete inversion, the placental area being the paralyzed portion, and sinking downward either from spontaneous or artificial causes. Yet, even in these cases, it can easily be understood how the shock thus produced, together with hæmorrhage, might subsequently cause general relaxation and allow further descent of the fundus.

DR. MERRIMAN was very much interested in the case, which had been reported in so clear and minute a manner. These dangers, although rare, were liable to occur at any time, and there was need to observe as closely as possible all circumstances connected with them, that the causes of the trouble might be more perfectly understood. He did not remember having seen an explanation offered for the first step in the process of inversion, namely, that of depression, except: 1st, the presence of a polypus; 2d, drawing upon an adherent placenta; 3d, pressure from above; but these were evidently not the only causes. Dr. Barnes gives as a circumstance frequently connected with this difficulty, the attachment of the placenta to the fundus uteri. Since the placenta undergoes absolutely no detachment during the strong contractile efforts of the uterus during labor, it seems probable that the placental seat is less affected generally by uterine contraction. If, then, the attachment should be at the fundus uteri, and an unusually relaxed state of the uterus exist, a very little pressure would be sufficient to start a depression which, once started, would readily go on from the contractile efforts of the uterus itself.

He thought of one other explanation. If irregular contractions should occur, they might affect only the external fibers of the uterine fundus, and it would readily be seen that this circumstance would cause the wall of the uterus to bulge inward, thus producing the initial step in the process of inversion. He thought, in this connection, that the effect of ergot, which is so liable to produce irregular contractions of the uterus, should be carefully watched.

DR. NELSON: It has not been my fortune yet to see a case of puerperal inversion of the uterus, but I can readily understand that the piston-like action of the placenta, referred to by Dr. Miller, might be, in a relaxed condition of the uterine walls, a cause of partial or complete inversion, if the cord were drawn upon while the uterus was thus relaxed. For this reason I make it a rule never to attempt to remove the placenta until the uterus is contracting firmly. Then, if gentle traction on the cord, or the presenting portion of the placenta, does not readily extract it, and especially if it seems to be firmly held, while the fœtal surface presenting at the os shows that it is at least partly detached, I bore through the presenting placental mass with the forefinger, to allow the air to pass through the placenta, and thus destroy this sucker-like action, and then the contents of the uterus may be readily removed. But until this is done, or in some way the air allowed to get behind the placenta, traction upon it or the cord will simply drag downward the uterus and its contents until the uterine contractions excited thereby expel the contents. I do not recollect to have seen this procedure described in any of the books, but I have, for several years, found it to greatly facilitate the removal of the placenta.

Dr. Sawyer's exact description of this case suggests also another possible cause of inversion, when the uterine walls are distended and relaxed, namely, pressure upon the uterus through the abdomninal walls by an assistant, especially if unskilled. For I well remember, in assisting Dr. Roler in a footling case, under ether, while pressing firmly upon the fundus of the uterus to hasten the delivery of the head and so save the child, I distinctly felt the walls of the uterus dimple under the pressure, especially when too great force was momentarily exerted by the thumbs and finger points in attempting to relieve the hand, and while the uterus was not firmly contracting. Indeed, the inertia of the uterine walls was the very reason for using these vigorous external means to hasten delivery.

Dr. Sawyer describes the compression of the aorta from the outside, which seems to have been satisfactory in his case; but, if the abdominal walls contain much adipose tissue, it might be more difficult. Following the teachings of my preceptor, Dr. Storer, of Boston, I should prefer to pass the hand directly into the uterus, and compress the aorta through the thin uterine walls. I have not found it difficult to introduce the hand into the uterus immediately after delivery, and the aorta can certainly be as safely compressed in this way upon the spine. Furthermore, the hand itself, in the uterus, will be a powerful means of exciting the uterine contraction, and should any portion of the walls be inverting, it would be in the best possible position to detect and replace it. It is also in position to detach the placenta, if this may have been the cause of the hæmorrhage, or to remove clots resulting from the hæmorrhage. I have had occasion to compress the aorta in this way but once, and saw no bad results.

As the tetanic contraction of the uterus was described in Dr. Sawyer's case following the use of ergot, the query came to mind, will the use of ether, carried to complete anæsthesia, in such cases, overcome the action of the ergot?

I would also like to ask the members of the society what their experience is in the use of the corn ergot—the fluid extract of ustilago maidis; will it produce, in full doses, these same tetanic contractions which the rye ergot frequently does? I have used the fluid extract of ustilago in my obstetric practice during the past year and a half, using it as I would the rye ergot, in doses of half a teaspoonful and more, and I have not yet seen the severe contractions occasionally produced by the rye ergot; neither is it as liable to disturb the stomach as that—perhaps because the taste is usually not as disagreeable to the patient.

DR. JONES said that he had little to add to what had already been said as to the etiology of inverted uterus.

During the discussion, it had occurred to him that possibly, as in early abortions, a certain balooning of the upper vaginal and lower uterine ligamentous structures might occur after parturition, which would draw apart the inert walls of the womb, and suffer the flaccid fundus and upper segment of the organ to fall into the cavity thus made. Then, under the stimulus of invagination, active contraction, grasping the inverted portion, might be set up, which would tend to complete the "perversion," or render it impossible for the attendant to effect its reinstatement. It was possible that the ergot administered in the case reported might have proved a disadvantage, but that it had anything to do with the causation of the accident, he could not believe. He agreed with other speakers in thinking inversion due mainly to uterine inertia, and that the uterus was not primarily active.

As for the commonly assigned cause—traction upon the funis—he averred his constant practice of that forbidden means of delivering the placenta, but always with the premise of normal uterine contraction. A case of puerperal inversion of the uterus had never occurred in his own practice.

DR. FITCH: I regret exceedingly that I did not arrive in time to hear the opening of this discussion, and the history of the case presented by Dr. Sawyer. My experience in puerperal inversion of the uterus has been very limited; indeed, in an active practice of twenty-seven years a case has never occurred under my own administration.

The only case I ever saw was in consultation. About twenty-two years ago, I was called to see a woman four or five days subsequent to her delivery. I found the patient of light complexion and frame, pale and haggard countenance, pulse 130 to 140, and feeble; skin cold, and covered with a clammy perspiration. Her labor was apparently normal. No excessive hæmorrhage had occurred, and no great degree of pain; yet there was constant vomiting, and great prostration. The physician in attendance had made no diagnosis, but supposed she had "child-bed fever."

I proceeded to make an examination per vaginam. The uterus was low in the pelvic cavity; os open to the size of a silver dollar, and a hard, somewhat roughened convex body presenting, which I at once suspected to be the fundus uteri. I immediately applied firm pressure with two fingers against this body, and counter pressure upon the abdomen, for the space of five minutes; the body gradually receded from the os, and suddenly left my fingers, with a decided thud; at the same instant the uterine tumor appeared in the hypogastria. The vomiting ceased at once; reaction quickly came on, and the patient made a good recovery.

In regard to the influence of ergot in the production of this accident, I believe that its action is upon the entire organ, producing uniform contraction of its muscular walls; therefore cannot favor the accident. My judgment is based on the use of it in probably 75 per cent. of all the cases I have attended during the past twenty years. I believe it is useful in assisting the delivery of the placenta; and further, by producing tonic contraction, it thoroughly empties the uterus, and prevents accumulation of blood in its cavities. For these purposes it is given from 10 to 20 minutes before the completion of the second stage of labor.

I have entertained the opinion that acute puerperal inversion was unnecessary; and that, when it did occur, it was usually due to neglect in the management of the delivery, or by too active interference.

To avoid this accident, as well as post-partum hæmorrhage, I am in the habit of pursuing the following course: As soon as the child's head is delivered, I direct the nurse, or other convenient person, to apply the hand to the hypogastrium, and follow the receding walls of the abdomen, with pressure directed upward and backward. The person is retained in that position till I have attended to the child, tied and severed the cord. I then place the patient on her back, and apply my free hand to the abdomen, over the uterus, grasping the organ with the thumb and two or three fingers, with the little finger resting on the fundus, for the purpose of promptly detecting any depression or dimpling. If the uterus does not contract promptly, I squeeze it forcibly. As soon as contraction occurs, I apply traction on the cord, equal to a weight of one half lb. to six lbs., at the same time pressing the lateral walls of the uterus, for two purposes: 1st, to assist in the expulsion of the placenta; 2d, to sustain these walls in apposition, and thus prevent inversion. Should dimpling be felt, I at once desist from traction; or should tearing be felt in the cord, traction should cease. I have never torn off a cord or inverted a uterus.

DR. ETHERIDGE remarked that the specimen before the society seemed an unusually large puerperal uterus.

DR. JACKSON had nothing to add to what had already been said in reference to inversion of the uterus. He had never been so unfortunate as to encounter a case in his own practice, and the specimen now before the society was the first he had ever seen. He only desired to express his surprise at the opposing views to which utterance had been given in reference to what should constitute the proper treatment of the third stage of labor, in its bearings upon the causation of the accident in question. One gentleman considered it dangerous to make pressure upon the fundus of the uterus in order to aid the expulsion of the placenta, while another thought this method the best and most favorable. Several regarded traction by the cord as highly dangerous, while others again, on the other hand, habitually delivered by pulling at the cord, and only limited the strength of the pull by the strength of the cord.

A number were in the habit of administering a dose of ergot to aid the expulsion of the placenta, while another was afraid to give it lest it should not be expelled at all.

Inversion of the womb was usually considered to be a very rare complication of labor; and yet, after listening to this discussion, it seemed wonderful that it was not a very common one. He thought the efficiency of some of the alleged causes was exaggerated. Certainly traction of the cord was. If it ever caused inversion, it must be only in exceptional cases; for he believed that fully 90 per cent. of all placentae were delivered by more or less traction by the funis. His own impression was, that puerperal inversion occurs usually from a passive, inert, relaxed condition of the uterine walls, produced by over-distension, as in the case described by Dr. Sawyer, or from some other causes.

DR. W. H. BYFORD believed that mere atony of the uterus was not a sufficient cause of inversion of the uterus. This condition might be a predisposing cause, and it was probably in this state of the womb that pulling on the cord and other injudicious efforts to deliver the placenta were rightly blamed as causes of inversion. He thought, however, that inversion more frequently resulted from irregular contraction of the uterus, in the sense that some of its fibers were acting strongly, while others were not contracting at all. In other words, there was partial, instead of general atony; one or more patches, limp and flabby, prolapse into some part of the globular arch of the contracting organ, and thus form the dimple so generally supposed to be necessary as a start toward inversion. The vehemence of contraction which this intruding portion would cause, is recognized by most writers as sufficient to complete the displacement. This state of partial atony must occupy fibers partially paralyzed by some circumstance existing during gestation, or occurring at the time of labor.

In the case of Dr. Sawyer, reported with so much intelligence and minuteness, the condition of the uterus anterior to delivery was sufficient to account for a weakened state of the fibers. There was a very considerable dropsy of the chorion, and the uterus contained an extraordinary amount of liquor amnii. This fact, interpreted, means that the fibers were *stretched.* In normal pregnancy the uterine fibers are not stretched; the whole organ is hypertrophied, and the actual addition of tissue, or growth of the muscles of the uterus, form a receptacle for the ovum that contracts uniformly and with vigor for the expulsion of its contents. Now, if an abnormal amount of fluid is secreted, it is accommodated not by a further increase of the muscular tissue forming the uterine globe, but the organ is *distended,* and the muscular wall becomes thinner by the distension. Thus stretched and thinned, for perhaps weeks before confinement, the fibers become weaker, and the labors succeeding this state of things are precisely the kind described by Dr. Sawyer. The contractions are painful and inefficient, the fibers acting discordantly until the fœtus is expelled or removed, as it was in this case. It is almost certain that the efficiency of some portions of the fibers is more impaired than that of others. It is well known that in uteri thus over-distended we have the hour-glass contraction — the incomplete contraction — accompanied by hæmorrhage, etc., and why not also inversion ?

If the ergot had any injurious influence in the reported case, it was not in causing the inversion, but in rendering the fibers more resistent to efforts at reposition. Dr. Byford further said that the treatment instituted by Dr. Sawyer was such as would suggest itself to most intelligent obstetricians.

It was quite apparent, however, from the tenor of the discussion to-night, as well as the diverse theories of inversion promulgated by writers on obstetrics, that there is yet much to be learned in reference to this most interesting subject.

With reference to the cause of death, he thought there could be no doubt but that it was due to shock, and the hæmorrhage was not sufficient to contribute materially to that end. In expressing this opinion, he would remind the society that the overwhelming influence of shock upon the nervous system depended as much upon the incapacity of certain persons to rally from nervous depression, as upon the force of the causing influence. What would kill some persons would not, on account of the inscrutable elasticity possessed by others, have any effect upon them.

DR. SAWYER: I feel indebted to the Fellows for the full and very intelligent discussion which my report has elicited, as well as for the very charitable willingness which has been shown to exonerate me from responsibility in the unfortunate termination of the case. The only point which was raised in my mind upon reflection, was concerning the administration of ergot; not that its use in any degree caused the inversion; there is not one fact which could suggest such a presumption, but that the condition of tonic spasm of the uterus, which prevented the immediate reduction of the displaced organ, might have been excited by the drug. I am assured, however, by the remarks which have been made, that such a spasm might have been induced by the presence of the fundus in the cavity of the uterus. In this they are confirmed by no less an authority than W. Tyler Smith, a reference to whom has been made in my report.

The remark made by Prof. Miller, viz: that the fundus may be indented by the tips of the fingers, when compression of the uterus is injudiciously made, I hold to be of paramount importance. In this connection I may be permitted to allude to the dangers attending Credé's method of expressing the placenta. In using this method, we are instructed to seize the uterus in both hands, and to squeeze out the after-birth. Practically one does not compress the uterus uniformly in all its axes; the abdominal parietes render the posterior surface of the organ inaccessible, and the tips of the fingers are forced to press upon the fundus. Now, under certain conditions, is it not reasonable to suppose that the paralyzed fundus may be indented in this manner? In fact, I have no question but that, in the case under discussion, the point of departure was in the compression which was used under my direction.

The plan of compressing the abdominal aorta from within the uterine cavity, alluded to by Dr. Nelson, has long found a place in the literature of the subject, and is spoken of by Cazeaux. When the uterus is large, the procedure has the advantage claimed for it, namely, that of quickly recognizing any descent of the fundus. But it is an agonizing operation. Moreover, in order to compress the artery, the hand must be carried above the fourth lumbar vertibra, or nearly to the umbilicus, and this interferes with the proper retraction of the uterus. On this account, I give preference to compression through the abdominal parietes.

Concerning the cause of puerperal inversion, all observers have remarked that unusual distension of the uterus, as exists in some cases of multiple pregnancy, and in dropsy of the amnion, is a condition which favors the accident. But upon the initial step in the process of inversion, the profession seems divided. One class of observers hold that it is a condition of irregular contraction of the uterus, whereby it is inverted; in other words, that the process is an active one in its very beginning. Prof. Byford has very clearly given us the *modus operandi* of this process, for which he has expressed his preference. It is especially dwelt upon by W. Tyler Smith, its author, who has likened the process to inversion and intussusception of the intestine, upon the one hand, and speaks of its relations to spasmodic rigidity of the os uteri, and so-called hour-glass contraction with retention of the placenta. It may be said, in support of the active process, that the virgin uterus has been known to invert itself by unceasing contraction of the organ.

Another class of observers believe the first step in the process to be passive, so far as the uterus is concerned, and that the fundus falls into the body by its own weight, or is pushed, or drawn downward through some external force.

I am impelled to accept the latter as correct. It is true that I can only speak upon the authority of a single case, but it will be conceded that the opportunity for observation was as good as it was rare. At a given moment, I felt the outlines of the symmetrical but flaccid uterus, its fundus reaching nearly to the umbilicus. Shortly afterward, the organ had contracted, and its fundus was gone. Now, what occurred in that short interval? Of course positive knowledge is precluded, because the fundus of the uterus cannot be inspected, and my hand was not in the cavity of the organ. Bnt of one thing I feel convinced, namely, that there was no contraction, partial or complete, of the uterus before the fundus had fallen. In reality, the recognition of the contraction and the discovery of the absence of the fundus were simultaneous. From this moment, however, till the last, the uterus was in a state of tonic spasm. Had the victim survived the shock, I can easily understand how this contraction may have propelled the fundus, and have made the inversion complete.

There is one other condition which I think can have an influence in determining this accident. Dr. Merriman has called our attention to a predisposing cause laid down by Barnes, namely, the location of the placenta at the fundus. I desire to ask the Fellows if it ever occurred to them that the location of the placenta at the fundus is extremely infrequent, quite as accidental, and, it may be, as rare as its location in the cervical zone? Perhaps in this fact Dr. Jackson, and others, may find one explanation of the great rarity of puerperal inversion of the uterus. I have many times determined the location of the placenta, through the uterine souffle, but have never yet found the area of the greatest intensity of the souffle to be at the highest part of the uterus. I regret that the location was not ascertained in the case before us.

The usual site of the placenta, in the right or left lateral half of the uterus, upon its anterior or posterior wall, I hold is not a matter of chance, but is determined by a condition of the uterus which obtains in the very earliest days of pregnancy.—You will recall that when the impregnated ovule reaches the cavity of the uterus, it finds the lining of the organ, the decidua, so swollen that the walls of the uterus are in apposition, and no cavity can be said to exist. Observation has shown, however, that the nearest approach to an unoccupied space within the uterus, is at the superior angles of the organ. When the ovum escapes the uterine opening of the Fallopian tube, it is not impelled to insinuate itself between the apposed deciduæ, but attaches itself naturally at the upper angle of the body, where, as we have just seen, there is the most room. It is the attachment of the ovum which determines the location of the placenta.

After the third month of pregnancy, the fundus of the uterus contributes most in forming the increasing cavity of the body. So disproportionate is this growth that, at the end of pregnancy, the Fallopian tubes seem to be attached to the uterus in its lower third, instead of near the superior point, as in the unimpregnated state. The placental site remains the same, and though it may increase in size by its own growth, it can never be carried upward by the gradual ascent of the fundus. I am aware that when the empty uterus contracts upon itself after delivery, the highest border of the uterus very nearly approaches the placental site; still then the greatest portion of the latter will be inferior to a line drawn between the uterine opening of the Fallopian tubes.

If my reasoning is well founded, the conservative design of nature must be acknowledged; for we are told by Barnes that a momentary paralysis of the placental site usually exists after labor, and Prof. Miller has said that the uterine tissue is thicker and heavier at that point. I cannot but think that this paralyzed, heavy area would very frequently descend from its normal plane, if the site were at the highest part of the uterus. But, grace to the fact that the site is mostly upon the side of the spheroid, its tendency is to fall outward.

I am not aware that these views have been expressed by others, but I have held and communicated them to my class for three years, and have confirmed the observation concerning the usual site of the placenta by many auscultatory examinations.

I desire again to thank the society for the interest which has been shown in the subject and specimen introduced by me.

